# Single-Cell RNA Sequencing in Lung Cancer: Revealing Phenotype Shaping of Stromal Cells in the Microenvironment

**DOI:** 10.3389/fimmu.2021.802080

**Published:** 2022-01-19

**Authors:** Jianhong Zhang, Chengyang Song, Ye Tian, Xueying Yang

**Affiliations:** Department of Thoracic Surgery, The Fourth Affiliated Hospital of China Medical University, Shenyang, China

**Keywords:** lung cancer, single-cell RNA sequencing, tumor microenvironment, heterogeneity, phenotype modeling

## Abstract

The lung tumor microenvironment, which is composed of heterogeneous cell populations, plays an important role in the progression of lung cancer and is closely related to therapeutic efficacy. Increasing evidence has shown that stromal components play a key role in regulating tumor invasion, metastasis and drug resistance. Therefore, a better understanding of stromal components in the tumor microenvironment is helpful for the diagnosis and treatment of lung cancer. Rapid advances in technology have brought our understanding of disease into the genetic era, and single-cell RNA sequencing has enabled us to describe gene expression profiles with unprecedented resolution, enabling quantitative analysis of gene expression at the single-cell level to reveal the correlations among heterogeneity, signaling pathways, drug resistance and microenvironment molding in lung cancer, which is important for the treatment of this disease. In this paper, several common single-cell RNA sequencing methods and their advantages and disadvantages are briefly introduced to provide a reference for selection of suitable methods. Furthermore, we review the latest progress of single-cell RNA sequencing in the study of stromal cells in the lung tumor microenvironment.

## Introduction

Tumors grow within a highly complex and dynamic local environment composed of immune cells, stromal cells, endothelial cells (ECs), and other noncellular components, such as secreted cytokines and extracellular matrix (1). This complex ecosystem is collectively termed the tumor microenvironment (TME) (2). The interactions of cells in the microenvironment, not only between tumor cells and stromal cells but also between stromal cells, results in a constantly changing TME during tumor progression, resulting in a dynamic process that has an important role in tumor development. Therefore, it is increasingly accepted that the TME can be a target for tumor therapy (3, 4). Unlike tumor cells, stromal cells in the TME are genetically stable and promote tumor growth through phenotypic changes, so they are potential therapeutic targets  (5). Intriguingly, despite the therapeutic importance of these cells, the *in situ* phenotype of stromal cells remains elusive. Therefore, phenotypic analysis of stromal cells in the TME is highly desirable and will lead to a deeper understanding of the TME, which will help advance the diagnosis and treatment of lung cancer.

Single-cell DNA sequencing can identify mutations throughout the genome of a single cell but cannot detect significant expression differences in heterogeneous cells in the TME. Single-cell RNA sequencing (scRNA-seq) not only allows us to detect cell-to-cell transcriptional heterogeneity at the single nucleotide level but also identifies gene expression heterogeneity between single cells, and it provides an unprecedented resolution for the study of stromal cells that are composed of heterogeneous and phenotypically different cell populations (6, 7). This technique allows precise quantitative analysis of the gene expression profiles of different kinds of stromal cells at the single-cell level, thus revealing the complexity of the molecular composition and the differences between them. This information, however, most likely cannot be obtained in bulk transcriptional sequencing due to the lack of sufficient resolution (8). Here, we review the current common scRNA-seq methods and their advantages and disadvantages as well as the latest progress of scRNA-seq in the study of stromal cells.

## A Brief Overview of scRNA-Seq

### Introduction to scRNA-Seq

ScRNA-seq is a leading technique for exploring the transcriptome of individual cells in sequenced samples and is a powerful tool for researching gene expression patterns. ScRNA-seq technology generally has several workflows: sample acquisition, cell dissociation, single cell capture, reverse transcription, cDNA amplification, library preparation, and sequencing and analysis (9).

Sample acquisition is the first step in scRNA-seq, and in addition to surgically excised tissue and biopsy specimens, embryos (10), human-derived tumor xenografts (11) and blood cells, including stem cells and fully differentiated lymphocytes (12, 13), can be used. Some recent studies have shown that the scRNA-seq data obtained from cryopreserved solid tissue or single-cell suspensions are equivalent to those obtained from fresh tissue. After cryopreservation, the TME complexity of a single-cell suspension and solid tissue is preserved, and in terms of gene expression, cryopreserved cell suspensions show a high correlation, indicating that cryopreservation has little effect on the results of downstream analysis (such as biological pathway enrichment)  (14–16).

The next step is dissociation and capture of the target cells. Cell dissociation is a critical step in sample preparation, especially in dense tissues and three-dimensional organ models, and it is usually performed under enzymatic conditions with gentle mechanical agitation to facilitate cell separation. Of note is the need to select the appropriate protein hydrolase and to strictly control the digestion time. Common single-cell isolation and capture techniques include limiting dilution, micromanipulation, fluorescence-activated cell sorting (FACS), laser capture microdissection (LCM), and microfluidics. Limiting dilution, which uses pipette dilution to separate individual cells, is used sparingly because of its low throughput and time-consuming protocol. Micromanipulation is a classic method for extracting cells from early embryos or uncultured microorganisms (17), in which individual cells are manually selected under a microscope with a vitreous tube, and can be used for sampling from a limited number of cells or fragile cells, such as early embryos (18). Similarly, the method is low throughput, time consuming and technically demanding and is rarely used now. FACS is a common technique for isolating single cells with a high purity. Cells are first labeled with fluorescent monoclonal antibodies that can recognize specific surface markers and are capable of sorting different groups. FACS is also a preferred method when the target cells express very low levels of markers, but it is difficult to isolate samples with cell counts below 10,000 (19). LCM involves focusing a laser beam on cells of interest under a microscope, attaching these individual cells to a transparent membrane (20) and then transferring the single cell on the membrane to a microcentrifuge tube containing an appropriate buffer solution (21). This technique provides spatial information about the target cells, but the disadvantages are similar to those of FACS. Microfluidics refers to a technique that uses microscale structures to precisely control the supervolume of fluid and is currently a common method for single-cell capture. For example, the Fluidigm C1 microfluidic robotic platform or droplet-based microfluidics methods have been used to capture cells (22), and although this method has high throughput and a low analytical cost, the capture efficiency in viscous cells may be relatively low. A more detailed description of the cell separation capture method was recently described (23).

**Figure 1:**
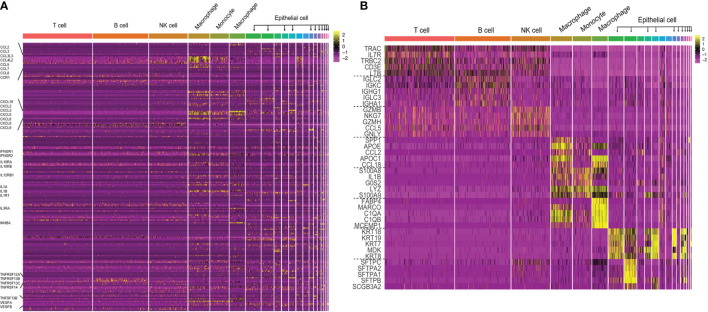
**(A)** The cytokine expression across different cell types in TME of LUAD. Performed an integrative analysis using Seurat v3 in RStudio by combining the scRNAseq data from multiple datasets in LUAD (GSE97168, GSE131907, GSE99254) and plotted the expression levels of 224 cytokines across different cell types in TME. **(B)** Heatmap of canonical cell-type markers of different cell types in TME of LUAD. Using the same datasets and strategy employed in the above cytokine analysis, generated a heatmap of canonical cell-type markers of stromal cells in TME of LUAD.

Many scRNA-seq methods have been developed, such as Massively parallel RNA single-cell sequencing (MARS-seq), Single-cell tagged reverse transcription sequencing (STRT-seq), Switching mechanism at the end of the 5´-end of the RNA transcript sequencing 2 (Smart-seq2), Massively parallel RNA single-cell sequencing (CEL-seq), Indexing droplets (InDrop**)** and droplet-based scRNA-seq (Drop-seq). These techniques mainly differ in the method of amplifying mRNA transcripts. Among them, Drop-seq has the lowest cost for analyzing a large number of single cells, Smart-seq2 sequencing has the highest sensitivity, and droplet-based 10× Genomics Chromium is the most widely used commercial platform. However, it is still necessary to choose the appropriate scRNA-seq method according to the actual situation of the experiment. More information on scRNA-seq has been summarized (**Table 1**) and can provide us with a reference in the selection process. In the subsequent steps of library construction and data analysis, scRNA-seq is based on essentially the same principles and processes as bulk sequencing. One of the more important things is cellular annotation, which needs to be done by integrating differentially expressed genes between clusters, expression of classical marker genes and enrichment of the reference gene set of immune cells in the literature. We combined scRNAseq data from multiple datasets in LUAD [GSE97168 (34), GSE131907 (35), GSE99254 (36)] to map the expression levels of 224 cytokines in different cell types in TME (**Figure 1A**), and specific expression patterns of several representative cytokines in certain cell types were highlighted (**Figure 1B**) for cell annotation. Analyzing scRNA-seq data is challenging because it requires multidisciplinary knowledge. Although more complex, this method can provide us with higher resolution and a deeper understanding of the lung cancer microenvironment.

**Table 1:** Commonly used single-cell sequencing methods and their advantages and disadvantages.

Method	Region	Unique molecular identifier (UMI)	Amplification	Advantages	Disadvantages	Depth	Reference
CEL-seq	3’-end only	Yes	*In vitro* transcription	Strand specificity (with more than 98% of exon reads coming from the sense strand), high barcoding efficiency (>96%), lower technical noise	The 3-bias and the low sensitivity for poorly expressed transcripts, high cost	10^4–10^5	(24)
InDrop	3’-end only	Yes	*In vitro* transcription	Sequence cells from heterogeneous populations quickly, identifying rare cell types	The very low capture efficiency (only 7%)	10^4–10^5	(25, 26)
Drop-seq	3’-end only	Yes	Template switching-based PCR	Rapid, low cost and high capture efficiency	Requires a cell suspension, only the 3’-most terminal fragments can be used for sequencing	10^4–10^5	(26, 27)
Smart-seq2	Full-length	No	Template switching-based PCR	Entirely relies on off-the-shelf reagents, high sensitivity	Samples can be pooled just prior to sequencing, more labor-intensive	10^6	(28, 29)
MARS-seq	3’-end only	Yes	*In vitro* transcription	A dramatic increase in throughput and reproducibility, increases the information content of the sampled transcriptional states	Higher median dropout probability	10^4–10^5	(30, 31)
STRT-Seq	5’-end only	Yes	Template switching-based PCR	Counting the number of unique transcripts expressed in each cell and tell them apart from PCR duplicates	The impossibility of detecting SNPs or splice variants located outside the 5’-terminal portion of the transcript	10^4–10^5	(32, 33)

**Figure 2:**
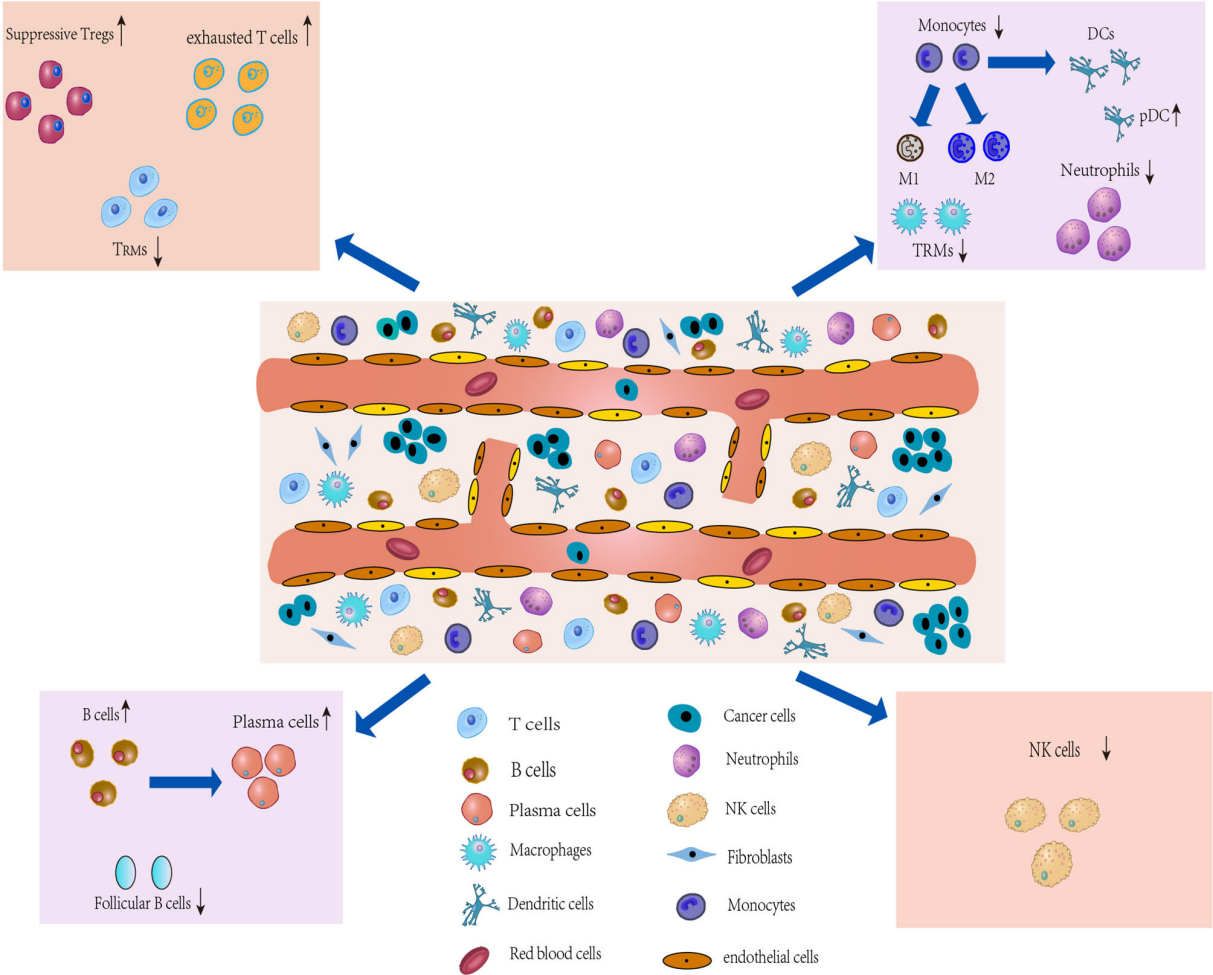
Characterization of the TME and development of IICs. The TME of lung cancer consists of multiple heterogeneous cell types, such as T cells, tumorassociatedmacrophages (TAMs), an CAFs. The number and phenotype of IICs change in the TME, and differential expression occurs. These different types of cells in the TME, along with lung cancer cells, drive the microenvironment toward immunosuppression.

### ScRNA-Seq and Spatial Information

Both temporal and spatial heterogeneity are the result of tumor progression (37). scRNA-seq, although able to identify cell subpopulations within tissues, their spatial distribution cannot be identified, nor can it capture local networks of intercellular communication (38). In contrast, spatial transcriptome is able to obtain different cell subpopulations and gene expression status in different regions of the tissue. Therefore, many spatial transcriptome technologies have been developed rapidly and become a frontier hotspot in biotechnology research in recent years. Currently, spatial transcriptome technologies are broadly classified into 4 types: microdissection-based, *in situ* hybridization (ISH), *in situ* sequencing (ISS), and microarray technologies (39). While most existing technologies can resolve to a few cells, single cell and subcellular resolution is being developed (39), and the quest to achieve a balance of a broad transcriptome, high resolution, and high gene detection efficiency has become the direction of spatial transcriptome development.

Due to the loss of spatial information in scRNA-seq, if scRNA-seq data can be matched one-by-one with information on their spatial location in tissues, it will help to understand the structure of cell type distribution and the putative mechanisms of intercellular communication that constitute this structure. Therefore, it is a good choice to integrate scRNA-seq data and spatial transcriptome data. Currently, there are two main methods: Deconvolution and Mapping (40). Deconvolution aims to separate discrete subpopulations of cells from a mixture of mRNA transcripts from each capture site based on single-cell data (40). Mapping has two facets: mapping of assigned scRNA-based cell subpopulations to each cell on high-plex RNA imaging (HPRI) maps and mapping each scRNA-seq cell to a specific niche or region of a tissue (40). This analysis can give a context for putative ligand-receptor interactions obtained from the analysis of scRNA-seq data. More detailed information about the integration of the two has been specified in a recent paper (40), and this will be the way forward. Multi-regional scRNA-seq has also shown good applications in spatial information of lung cancer (41–43). The further exploration of spatial information of lung cancer gives us new insights into tumor heterogeneity, tumor diagnosis and treatment.

## Phenotypic Molding of Stromal Cells in the Lung Cancer TME Under scRNA-Seq Analysis

The TME, characterized by heterogeneity, plasticity and complex cross-interactions, not only plays an important role in tumor development but also has a far-reaching impact on therapeutic effects (44). In addition to cancer cells in TME, tumor-associated stromal cells, which are composed of infiltrating immune cells (IICs), cancer-associated fibroblasts (CAFs) and ECs (**Figure 2**) (4), are also an important part and a key contributor to the TME (45). During tumor progression, the tumor cell “seeds” co-evolve with the surrounding microenvironment “soil” (4), driving the TME towards heterogeneity and immunosuppression. Heterogeneity is defined by the density, location and organization of stromal cell types and cytokines in the TME (46). Cancer cells can shape their TME by secreting various cytokines, chemokines and other factors, which leads to phenotypic changes in stromal cells in the TME (47) that may underlie stromal cell heterogeneity. In turn, immune cells produce an immune response against the tumor to shape the microenvironment. Similarly, stromal cells show heterogeneous expression and play a decisive role in tumor survival and progression by producing various growth factors, chemokines and cytokines that promote extracellular matrix remodeling, cell migration, neoangiogenesis and invasion in the TME (4), which will ultimately produce an immunosuppressive TME that promotes tumor growth and metastasis. Thus, the immunosuppressive TME is largely attributable to factors released by tumor cells that directly or indirectly bias the function and phenotype of IICs, CAFs, and ECs. While analysis of the TME can reveal relevant induced factors for protumor progression, and these factors are expected to contribute to the development of new tumor treatment strategies (48).

**Figure 3:**
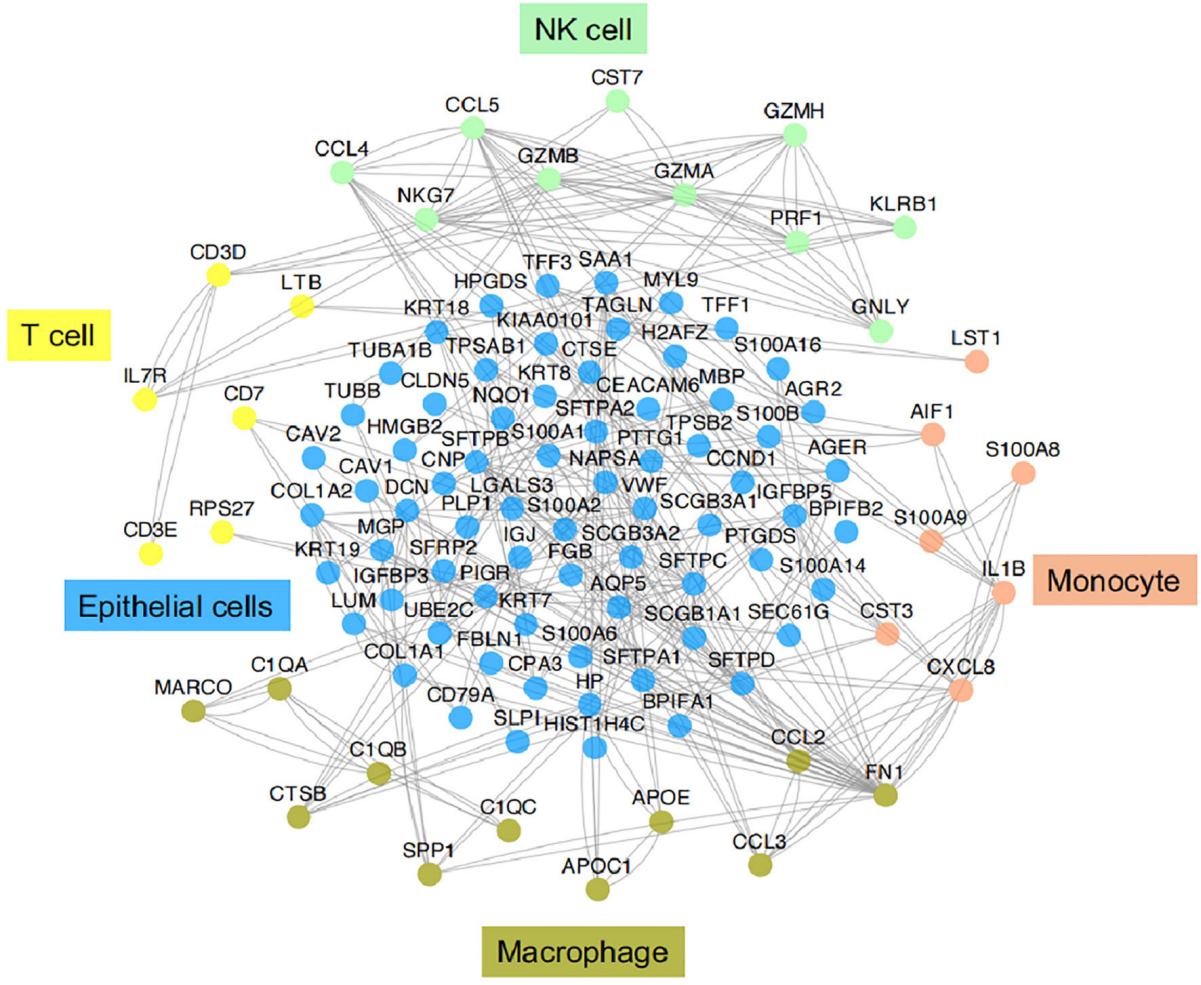
Functional association networks of signature genes between different cell types of IICs in TME of LUAD. Using StringDB with protein-protein interaction data showed functional association networks between signature genes specific to stromal cell of TME in LUAD.

Although the lung cancer ecosystem is highly complex, scRNA-seq technology has become a powerful tool for dissecting the TME of lung cancer by characterizing various cells, thus revealing their phenotypic changes and expression differences in the TME (49). Recently, studies on scRNA-seq in the TME of lung cancer have proliferated, and lung tumor cells have been specifically described in several recent papers (50, 51). However, the TME of stromal cells have not been described in detail. Here, we discuss stromal cells from the TME with measuring the transcriptome at the single-cell level provides an unprecedented perspective on cellular structure, showing phenotypic variations and functional differences in stromal cells of the TME (52).

### IICs

**Figure 4:**
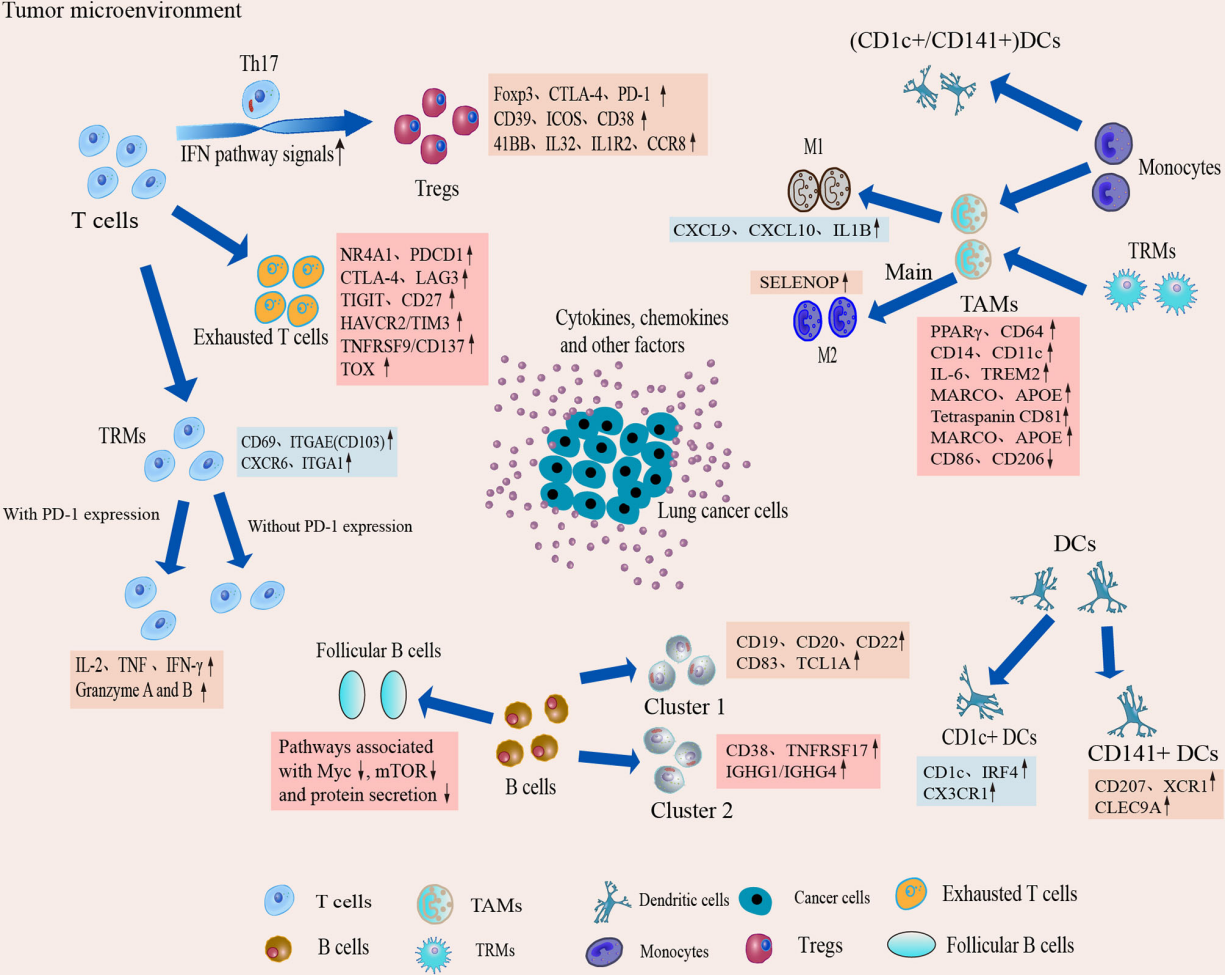
Dynamic changes and transcriptional characteristics of IICs in the TME. Tumor-infiltrating T cells undergo phenotypic transitions and functional state shifts that drive the immunosuppressive microenvironment. Macrophages are predominantly differentiated from monocytes in the TME, and TME sculpts them into animmunosuppressive phenotype, leading to accumulation of suppressive TAM and a tendency to differentiate toward M2. Single-cell interrogation helps identify novel subpopulations of TAM in TME and reveals that TAM subtypes tend to co-express M1 and M2 features. B cells play an important role in antitumor immunity and ICI therapy, but the B cells show heterogeneous subpopulations and reduced follicular B cell function in the TME. Two classical subgroups of DCs also show heterogeneous expression in the TME, which suggests that DCs can induce immune tolerance in the microenvironment.

IICs, an important part of the TME, have been shown to contribute to tumor progression and the immunotherapeutic response (53). These cells can interact with tumor cells to influence tumor progression and are a major target of current immunotherapies. It is well known that IICs exhibit significant heterogeneity and differentiation to different phenotypes in the TME. In TME, different subtypes of cells perform their own functions, which in turn are closely related to the genes or pathways they express. Here we provide functional association networks between signature genes specific to stromal cells of TME in LAUD (**Figure 3**), revealing the functional crosstalk between cells in the microenvironment. Through scRNA-seq, we can understand the single-cell characterization, the phenotypic diversity and transcriptional dynamics of IICs (**Figure 4**), which is essential for deciphering the mechanisms of immunotherapy, defining predictive biomarkers and identifying new therapeutic targets.

**Figure 5:**
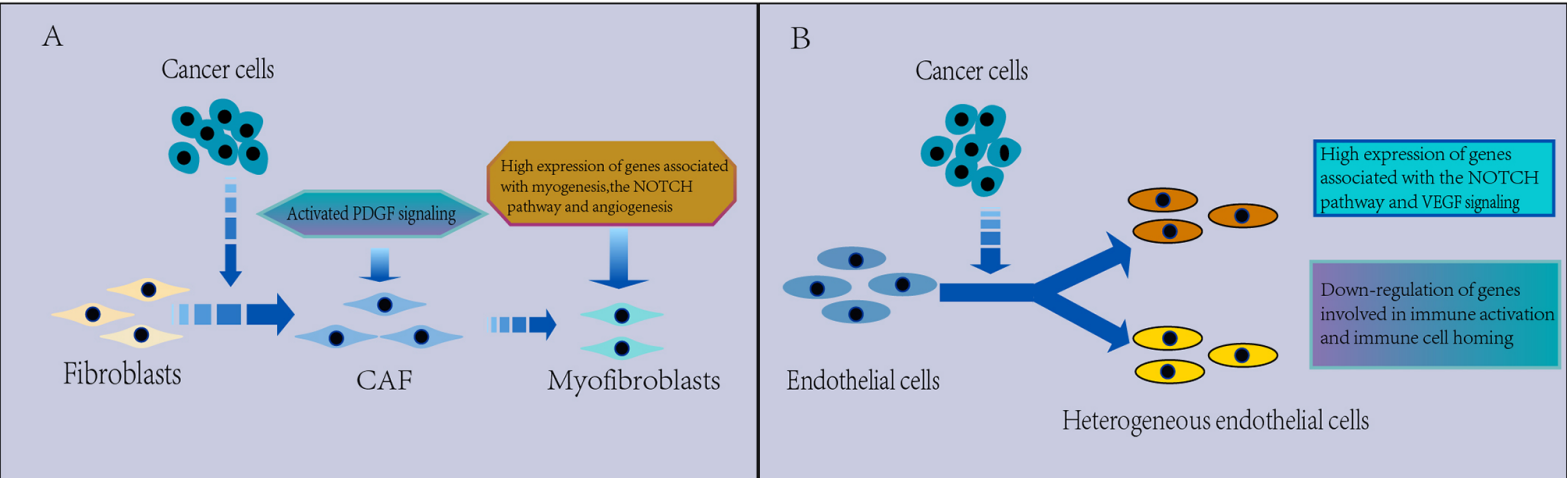
**(A)** Characterization of CAFs in the TME. CAFs are heterogeneous. Myofibroblasts were found to replace fibroblasts in the TME, which have a protumorigenic effect, with high expression of genes related to myogenesis, the Notch pathway and angiogenesis, and PDGF signaling is activated in the TME, which may be associated with the promotion of angiogenesis. **(B)** Characterization of ECs in the TME. The EC phenotype changes in the TME and appears significantly heterogeneous, which leads to tumor immunity toward immunosuppression. There is a strong activation of VEGF and Notch signaling that regulates EC development in NSCLC and downregulated expression of genes involved in immune activation and immune cell homing in tumor ECs, thus contributing to tumor immunetolerance.

#### Phenotypic Molding of T Cells in the TME of Lung Cancer

As a key component of adaptive immunity, T cells are a core of immunotherapy. However, they develop different phenotypes after chronic stimulation and interaction with tumor cells, forming distinct subpopulations. The heterogeneity of T cells can be revealed by scRNA-seq analysis. In the TME, scRNA-seq revealed that T cells were clustered into multiple clusters, containing several known types as well as multiple CD8^+^ T cell and CD4^+^ T cell clusters, and also identified several previously undescribed tumor-associated clusters in NSCLC (36, 54, 55). These results indicate the widespread heterogeneity of tumor-infiltrating T cells in NSCLC. There was also significant spatial heterogeneity of T cells in the TME. Applying the activation status of specific pathways or the relative abundance of specific immune cell types to define hot and cold immune profiles, Jia et al. found that eight genes associated with CD8^+^ T cell infiltration and cytotoxicity were significantly more highly expressed in the hot area than in the cold area (56). While the abundance of activated CD4^+^ T cells was not statistically different in these two regions, but the remaining 25 parameters representing immunogenicity were substantially inflamed in the hot area (56). The result suggests multifocal heterogeneity of T cells in the TME, and hot areas may have better immunotherapeutic effects. In addition, Li et al. found extensive isoform switching during T cell clonal expansion (57), suggesting that tumor-infiltrating T cells have strong phenotypic heterogeneity, and isoform information should be considered as a factor that may affect the efficacy of current immunotherapies. However, although T cells have different states, it is a gradual, rather than a discrete, state, as found in the pseudotime trajectory analysis of T cells in the TME by scRNA-seq combined with T cell expression patterns  (58).

The phenotypic shaping of T cells causes changes in T cell function, which may drive tumor immunity toward immunosuppression. In early lung adenocarcinoma (LUAD), T lymphocytes are one of the most abundant immune cells at the primary tumor site identified by scRNA-seq (34, 43), and these cells are increased compared to those in normal lung tissue (43), suggesting that adaptive immune responses are activated. However, more evidence indicates T cell phenotypic changes toward immunosuppression in the early LUAD microenvironment (59, 60). For example, regulatory T cells (Tregs) are enriched in primary tumor tissue, and the proportion of CD8^+^ T cells is reduced (34), while regulatory and depletion markers expressed by tumor-infiltrating T cells, such as TIGIT, LAYN, and CTLA-4, are significantly elevated in EGFR-mutated early LUAD (61). The changes in cellular composition and gene expression phenotype of T cells confirmed the direction of tumor immunity toward immune suppression in LUAD. However, the suppressive tendency was not limited to early lung cancer, and a significant increase in the proportion of Tregs and exhausted CD8^+^ T cells was found during LUAD progression and metastasis *via* scRNA-seq (43). Exhausted CD8^+^ T cell clusters were specifically enriched in NSCLC, and the abundance of exhausted CD8^+^ T cells in tumors was positively correlated with disease progression (62), suggesting that the immunosuppressive tendency of tumor immunity persists and gradually strengthens during lung cancer progression. However, researchers notably found that all innate immune changes shown in the advanced stage were present in early-stage lesions (34). This finding suggests that dysfunction of innate immunity may play a critical role in tumor development and that reinvigorating innate immunity against tumors will be a potential strategy to block tumor formation and progression.

Different tumor-infiltrating T cell clusters may have different phenotypic and functional properties. Although the tumor-infiltrating T cell clusters defined by scRNA-seq analysis vary with tumor origin, type, and disease stage, several major tumor CD4^+^ and CD8^+^ T cell populations with different phenotypes are broadly consistent in the identification of different types of NSCLC, including exhausted T cells, tissue-resident memory T (T_RM_) cells, and Tregs. These populations have important roles in antitumor immunity, and scRNA-seq technology has facilitated a better understanding of these cells in the TME.

##### Tregs

Tregs are a subpopulation of T cells that control autoimmune responses *in vivo*. Tregs are involved in tumor immunity and can be converted from CD4^+^ T cells. The proportion of CD4^+^ Tregs is elevated in all types of cancer, and studies have shown that the expansion of the Treg population in tumors is due to phenotypic switching rather than increased proliferation of preexisting Tregs (41, 63–65). CD4^+^ Tregs negatively regulate the immune response against tumors, and an increased proportion implies weakened antitumor immunity (66). Therefore, it is important to apply scRNA-seq technology to research CD4^+^ Tregs in the TME.

Classic CD4^+^ Tregs are characterized by the expression of the transcription factor forkhead box protein P3 (FOXP3). However, several recent studies have found that FOXP3- CD4^+^ T cells also show suppressive immune responses in the TME (67, 68), displaying similar functions as FOXP3^+^ CD4^+^ Tregs. In order to find out the reason for it, application of the Chromium Single Cell 3′ protocol to CD4^+^ T cell analysis identified EOMES^+^ CD4^+^ T cell subsets that play a suppressive role in FoxP3- CD4^+^ T cells in NSCLC (69), demonstrating the unique advantage of scRNA-seq in finding specific small subsets.

ScRNA-seq analysis revealed that tumor-infiltrating Tregs have altered phenotypes and shown significantly heterogeneous compared to normal tissue. For example, scRNA-seq revealed restricted overlap between CD4^+^EOMES^+^ T cells with clonal expansion and the FOXP3^+^ Treg clonal subpopulation, suggesting the emergence of two phenotypically distinct subpopulations during phenotype shaping (69). Another study also showed a characteristic bimodal distribution of TNFRSF9 (a Treg activation marker), demonstrating that at least two subpopulations of Tregs exist (36). ScRNA-seq analysis revealed that the transformation of CD4^+^ T cells into Tregs is a continuous process associated with tumor cells. Through scRNA-seq, trajectory analysis in NSCLC showed a developmental pathway from naive CD4^+^ T cells to Tregs and identified a transitional phenotype, Th17-like cells, suggesting a potential interconversion of Th17-like cells to Tregs (70). In KRAS-mutated NSCLC, tumor-derived exosomes play a major role in the transformation of CD4^+^ T cells into Tregs (63), indicating that tumor cells can induce changes in immune cell phenotype through exosomes.

Tregs in TME exhibit specific expression, which may be a target for immunotherapy to reduce Tregs infiltration or inhibit their immunosuppressive activity, which may indirectly enhance anti-tumor T cell responses. ScRNA-seq revealed increased expression of Foxp3, CTLA-4, PD-1, CD39, ICOS and CD38 in Tregs in LUAD (34, 41), IL32 also showed higher expression in Treg cells 65, and these altered expressions may underlie its immunosuppressive effects. Infiltrating Tregs express specific signaling molecules on their surface, such as interleukin-1 receptor 2, programmed death (PD)-1 ligand 1, PD-1 ligand 2 and CCR8 chemokines (71), which have not been described previously, suggesting that tumor-infiltrating Tregs are highly suppressive and can regulate several immune checkpoints. In addition, interferon (IFN) pathway signaling was strongly shown during the phenotypic switch from CD4^+^ naïve cells to Treg-like cells, and upregulation of the expression of several IFN-stimulated genes occurred (63), indicating that IFN signaling is a driver of their phenotypic switch. These studies suggest that the application of scRNA-seq to lung cancer research may help us develop more meaningful new strategies in lung cancer treatment.

Tregs are characteristically distributed spatially in LUAD. Multi-region ScRNA-seq showed that LUAD tissues were particularly enriched with FOXP3^+^ Tregs. the cellular signature of Tregs became more pronounced with spatial proximity to LUAD (41). Notably, the fraction of Tregs co-expressing CTLA-4 and TIGIT immune checkpoints progressively increased along the spectrum of distant normal sites to more adjacent (to the tumor) regions up to the LUAD (41). In contrast, we noted a decrease in cytotoxic CD4^+^ cytotoxic T cell characterized by high expression of GZMA, GNLY or GZMA and GZMH, increasing proximity to LUAD (41).

##### Exhausted T Cells

Cytotoxic CD8^+^ T lymphocytes are considered to be the major T cell subtype that directly kills tumors. The cytotoxic CD8^+^ T cell phenotype is altered in the TME and is mainly in a dysfunctional and exhausted state. ScRNA-seq studies have shown a significant increase in the proportion of exhausted CD8^+^ T cells during LUAD progression and metastasis, while with increasing tumor proximity, CD8^+^ T cells was shown a gradual decrease, which were depleted in the LUAD (41, 43). It characterized by a lack of classic effector cytokine secretion and cytolytic function, as well as increased expression associated with T cell exhaustion, such as HLA-DRA (36, 65, 72). The states from cytotoxic CD8^+^ T cells to dysfunction and exhaustion exist in a continuum in NSCLC (73). Analysis of CD8^+^ T cell trajectories in the TME confirmed that the state of CD8^+^ T cells appears to be shaped by two distinct processes, an intrinsic T cell developmental program and a tumor-induced T cell depletion program (36). Therefore, intratumoral cytotoxic CD8^+^ T cells are not composed of discrete cell subtypes, but a continuum of cell states driven by ongoing activation and differentiation in response to TME stimuli.

Research on CD8^+^ T cells has focused on how to inhibit and reverse the phenotypic shift from activation to exhaustion. Therefore, the study of T cells in the pre-exhausted state *via* scRNA-seq is important and may lead to the identification of key factors that induce T cell exhaustion and optimize T cell-based lung cancer therapy. The expression of transcription factor NR4A1, which induces T cell exhaustion, was found to be upregulated in the late exhausted state through scRNA-seq (74), implying that NR4A1 may promote T cell exhaustion. Under chronic antigen exposure, the expression of Tcf7 is associated with a sustained T cell response. The expression of Tcf7 in dysfunctional CD4 T and CD8 T cells analyzed by scRNA-seq and flow cytometry (75) showed that TCF7-expressing early differentiated T cells sustain immunity. In addition, dysfunctional T cells have distinct developmental and regulatory programs. Hypofunctional intratumor effector T cells are marked by the expression of the inhibitory coreceptors TOX51 and CD39, and the expression of genes encoding costimulatory and inhibitory receptors is inconsistent among different dysfunctional subpopulations, implying that potential immunotherapeutic targets are differentially regulated in different dysfunctional subpopulations (75). There is also a relationship between pre-exhausted T cells and prognosis. ScRNA-seq of T cells isolated from tumors, adjacent normal tissue and peripheral blood showed that the ratio of “pre-exhausted” T cells to exhausted T cells was associated with a good prognosis in LUAD. No such trend was observed in lung squamous cell carcinoma (LUSC) (36). This finding suggests a potential difference between lung cancer types, and the reason needs to be further investigated.

In addition to understand T cells in a pre-exhausted state, the study of phenotype conversion to exhausted T cells in the TME is equally important. Through scRNA-seq, tumor-derived CD8^+^ T cells exhibited specific expression in NSCLC. Lambrechts observed that the cell proliferative capacity of tumor-derived T cells was low, but a highly proliferative cluster and two clusters with high allograft rejection activities were identified in CD8^+^ T cells (54), which may be related to the fact that cells show higher responsiveness to the new epitopes encoded by cancer cells. These cells express higher levels of immune checkpoint molecules, including the approved targets PDCD1 and CTLA-4 but also other targets currently in clinical trials (LAG3, TIGIT, HAVCR2/TIM3, CD27 and TNFRSF9/CD137), and higher cytotoxic activity was found to be inhibited by these factors (54). In another study, exhausted CD8^+^ T cells in NSCLC were enriched with co-suppressed immune checkpoints, including CTLA-4 and TIGIT (70), and these molecules may be used as targets for immune checkpoint inhibitor (ICI) therapy. Some of them have already started to be used in clinical treatment with good therapeutic results, such as CTLA-4 (76). In addition, thymocyte selection-associated high mobility group box gene (TOX) expression was upregulated along the pseudotime of CD8^+^ T cell exhaustion by scRNA-seq, and TOX promotes intratumoral CD8^+^ T cell exhaustion by upregulating the expression of immune checkpoint (IC) molecules (74), suggesting that TOX inhibition may impede T cell exhaustion and improve the efficacy of ICIs. In LUAD, granzyme B expression in CD8^+^ T cells was significantly reduced upon stimulation (34), indicating that CD8^+^ T cell cytotoxicity was impaired. The differential expression of impaired CD8^+^ T cell toxicity is also related to the spatial, with CD8^+^ T cells exhibiting significant and spatially-modulated reduction in cytotoxic signature scores and decreased expression of major cytotoxic genes, including reduced levels of NKG7 and GNLY expression *via* multi-region ScRNA-seq (41). Surprisingly, however, exhausted CD8 T^+^ cells also express genes associated with conventional CD8^+^ T cell effector function and sustained proliferation, but at a lower level than fully activated effector cells. suggesting that activation of exhausted CD8^+^ T cells may be an option to inhibit tumor growth (41).

##### T_RM_s

T_RM_s are memory CD4^+^ or CD8^+^ T cells retained in peripheral tissues and patrol areas other than lymphoid organs to defend against pathogenic infections (77). T_RM_s permanently reside in tissues expressing CD69 and CD103 (78) and provide local protective immune responses. In some solid tumors, T_RM_s may play an important role in controlling tumor growth and limiting cancer cell metastasis (79). High T_RM_ infiltration is associated with better survival in lung cancer patients (80).

Several scRNA-seq studies have shown that T_RM_ subpopulations are heterogeneous in NSCLC, exhibiting different phenotypes and gene expression patterns (36, 81). For example, scRNA-seq analysis of T_RM_s in lung cancer identified five previously undescribed clusters of cells with different phenotypes, and cells in different clusters have different gene expression programs (81). T_RM_ marker genes, such as CD69, ITGAE (CD103), CXCR6 and ITGA1, are overexpressed in tumor-associated T cells in general but show different expression patterns in different subgroups (36).

PD-1 expression is considered a typical feature of exhaustion (82), while some recent studies found that high PD-1 expression reflects tissue residence rather than exhaustion (83, 84). ScRNA-seq analysis of T_RM_s revealed that T_RM_s expressing PD-1 had increased expression of the key effector cytokines IL-2, tumor necrosis factor (TNF) and IFN-γ, as well as increased expression of the cytotoxic molecules granzyme A and granzyme B compared to T_RM_s without PD-1 expression in NSCLC (81), suggesting that PD-1 expression does not reflect exhaustion. PD-1 expression levels were confirmed to be higher in lung cancer and lung tissue T_RM_s than in non-T_RM_s at the protein level (83), suggesting that the high expression of PD-1 may reflect tissue residency.

ZNF683 encodes a transcription factor involved in T_RM_ generation and maintenance. Comprehensive analysis of T cells in combination with scRNA-seq data showed that the frequency of ZNF683^+^CD8^+^ T_RM_ cells was significantly higher in NSCLC tumors than in other tumors (36). Based on recent scRNA-seq data and bulk RNA-seq data from TCGA, it was found that the higher the ratio of T_RM_ to exhausted T cell signature gene expression, the higher the overall survival rate of NSCLC patients was (81). These scRNA-seq studies indicate an important role of T_RM_s in solid tumor control.

#### Phenotypic Shaping of B Cells in the TME of Lung Cancer

As an important component of adaptive immunity, B cells mediate antitumor immune responses in the TME. It has been shown that the increase of B cells in TME contributes to the establishment of Tertiary lymphatic structure (TLS), which provides a harbor for lymphocyte maturation and immune activation (85). In TLS, T cells and B cells can undergo synergistic maturation, activation and clonal expansion, and thus B cells can promote responsiveness to immunotherapy for lung cancer (85). Through scRNA-seq, B cells were found to be highly enriched in both early- and late-stage NSCLC (41, 43, 54, 86), which indicates that the humoral immune response is activated.

The heterogeneity of B cells has begun to be revealed by several scRNA-seq studies. Lambrechts found that B cells were clustered in multiple clusters *via* scRNA-seq, and the ratio of different B cell subpopulations differed between tumor and normal tissues in NSCLC (54). Kim found that different B cell subtypes exhibited slightly different variable gene expre0. 3ssion profiles (43), suggesting the generation and clonal proliferation of tumor antigen-specific B cells. Both studies found reduced follicular B cell function (43, 54). Tumor-associated follicular B cells showed decreased oxidative phosphorylation, cell proliferation, biomass production (pathways related to Myc, mTOR and protein secretion) and reduced number of transcripts compared to follicular B cells in normal lung tissue, suggesting that follicular B cells become exhausted in the NSCLC (54). In addition, expression profiling of plasma cells revealed spatial differences in isotype-switching, such as increased IGHA1/2 and decreased IGHG1/3 with increasing proximity to LUAD (41).

CD20 and CD19 are the most commonly used markers of B cells, and studies have found that CD20 B cells may inhibit the growth and progression of tumor cells in the early stages of the disease and are positively associated with a good prognosis in NSCLC. ScRNA-seq study on NSCLC tumors and blood B cells showed that B cells were divided into two major subtypes: B cell cluster 1 expressed naive B cell markers (CD19, CD20, CD22, CD83, and TCL1A), B cell cluster 2 expressed plasma B cell markers (CD38, TNFRSF17, and IGHG1/IGHG4) (55), CD20-expressing naïve B cells are predominantly located in TLS, with a significantly lower proportion as the tumor progresses (55). CD20 B cells can directly inhibit NSCLC growth with good prognosis, and there is overexpression of secreted proteins, such as VNN2, IFI30, PIK3AP1 and SERPINA9, but their related functions need to be further explored (55). While in subpopulations of plasma-like B cells, IGHG1 and IGHG4 genes, which encode IgG proteins, were highly expressed, suggesting that the action of plasma-like B cells on tumor cells may be related to the secretion of IgG proteins in NSCLC (55), which may underlie the functional diversity of B cells, suggesting that targeting a specific subpopulation rather than the complete B cell population may be a future prospect.

#### Phenotypic Molding of Tumor-Infiltrating Myeloid Cells (TIMs) in the TME of Lung Cancer

Composed of macrophages, dendritic cells (DCs) and neutrophils, TIMs play a crucial role in tumor immunity (87). The TME contains many kinds of immune cells and targeting TIMs for immunotherapy in addition to T cells may be an effective strategy. TIMs were among the most abundant immune cells observed at the primary tumor site in LUAD *via* scRNA-seq, but in reduced abundance compared to normal lung tissue (43). TIMs are very complex with multiple lineages, and each of these lineages may further diversify into a spectrum of activation states in the presence of external stimuli (11, 54, 61). Thus, there is substantial phenotypic heterogeneity. Notably, metastatic lymph nodes in LUAD contained a large number of myeloid cells compared to normal lymph nodes, suggesting that bone marrow infiltration is associated with metastasis (43). However, it is not clear which subtype is specifically associated with.

##### Tumor-Associated Macrophages(TAMs)

There are two sources of macrophages: monocyte-derived macrophages (MDMs) and tissue-resident macrophages (TRMs). Macrophages are cellular components of the innate immune system and are present in almost all tissues. These cells play an important role in tissue homeostasis, immune monitoring and coordination of inflammation (88). TAMs show extremely high plasticity in the TME and are one of the major stromal cell types in the TME that promote tumor progression. Studies based on scRNA-seq may provide further insight.

ScRNA-seq analysis revealed the differentiation trajectory of macrophages in the TME, demonstrating that macrophages have significant heterogeneity and exhibit immunosuppressive activity in TME of lung cancer. Through scRNA-seq, multiple distinct phenotypic and expression characteristics of macrophage subpopulations were identified from lung cancer lesion areas compared to those from normal lung tissue in NSCLC (34, 86, 89), indicating extensive heterogeneity driven by both patient and tissue specificity (54). Through scRNA-seq, MDMs were found to be the main source of TAMs in primary lung tumors and distant metastases in LUAD, and their proportion increased significantly with tumor progression and metastasis (43, 89). Monocytes in tumors were identified in three differentiation tracks by applying the Monocle2 track analysis method. Monocytes can differentiate into inflammatory macrophages (M1 macrophages), monocyte-derived DCs (CD1c^+^ or CD141^+^ DCs) with antitumor immune functions, or alternatively activated macrophages (M2 macrophages) (86). It is suggested that macrophages distributed in the microenvironment are heterogeneous cell populations in different states, which may be responsible for their significant heterogeneity. In addition, scRNA-seq revealed the characteristics of macrophages in terms of spatial distribution. M2-like TAM is enriched in hypoxic and necrotic TME regions with limited antigen presentation capacity and abundant secretion of protumor factors (90). M2-like macrophages(CD163+) and monocytes were gradually depleted with increasing tumor proximity, whereas M2-like macrophages (TREM2+) proliferating myeloid subsets were steadily enriched in the tumors (41). In addition, the spatial distribution of PD-L1+ macrophages were analyzed in combination with scRNA-seq data, and they were found to accumulate in tight clusters at the tumor invasive margin (43). PD-L1+ macrophages can promote tumor progression, and anti-PD-L1 therapy was found to inhibit therapeutic growth by increasing macrophage phagocytosis (91). Spatial distribution of PD-L1+ macrophages suggest their possible association with tumor aggressiveness.

ScRNA-seq revealed that TAMs had a significantly different transcriptional profile compared to normal tissue-derived macrophages, exhibiting immunosuppressive activity. Paired mass cytometry (CyTOF) analysis of scRNA-seq data from early LUAD showed that TAMs expressed higher levels of the immunomodulatory transcription factor PPARγ, CD64, CD14 and CD11c and lower levels of CD86 and CD206. TAMs also expressed more IL-6 than intrapulmonary macrophages (34), and IL-6 is reported to have a potential effect on tumor aggressiveness (92). These results emphasize the immunosuppressive role of macrophages in early LUAD lesions. TAMs were also found higher expression levels of triggering receptor expressed on myeloid cells-2 (TREM2), tetraspanin CD81, macrophage receptor with collagenous structure (MARCO), and apolipoprotein E (APOE) in early LUAD (34). Based on the TCGA database, a significant survival disadvantage was observed in patients with a high percentage of these expression profiles. APOE was reported to promote tumor cell growth and aggressiveness (93), and was also found high expression in TAMs in another scRNA-seq study of early LUAD (61, 65). SPP1 and CCL2 were found high expression in LUAD through scRNA-seq (65), and they are also reported to promote tumor metastasis (94). It suggests that TAMs can promote lung cancer progression through inhibiting tumor immunity and promoting of tumor growth and invasion. Among the subtypes of TAMs, M2 macrophages were found high expression of SELENOP, whereas M1 macrophages expressed high levels of pro-inflammatory chemokines, such as CXCL9 and CXCL10, pro-inflammatory cytokine IL1B, and M1 macrophages were also found to be associated with other myeloid cell types such as plasmacytoid DCs in LUAD via scRNA-seq (95).

Although monocyte macrophages have multiple differentiation trajectories, TAMs transcriptional features indicate that polarization toward M2 is the main pathway of monocyte macrophage differentiation, and M1 was significantly associated with prognosis. Based on trajectory analysis of early NSCLC scRNA-seq data, the expression of known differentiation markers associated with macrophage M2 polarization (MRC1/CD206, MSR1/CD204, PPARG and TREM2) was upregulated, while the expression of proinflammatory cytokines (CXCL2 and IL1B) and transcription factors (JunB and NFKBIA) was downregulated in the transition state (86). Genes with downregulated expression are repressed for the differentiation of monocytes to the M2 phenotype, and their low expression indicates a tendency to differentiate toward M2 macrophages (86). Single-cell regulatory network inference and clustering (SIENIC) was applied to show that the expression of genes regulated by the IRF2, IRF7, IRF9 and STAT2 transcription factors was upregulated in TAM subpopulations, while the levels of genes regulated by Fos/Jun and IRF8 expression were decreased (54). Fos/Jun enhances the inflammatory response of macrophages, while IRF8 facilitates M1 polarization (96, 97). These results also support M2 polarization of TAMs and indicate the general presence of M2 macrophages in early lung cancer. However, in another study, TAMs were found to be moderately polarized, and macrophages in both tumor and normal lung tissues did not exhibit specific M1 or M2 features through scRNA-seq in early LUAD, suggesting no significant M1 and M2 polarization (61). The reasons for this finding need further investigation.

Although most studies on TAMs suggest that TAMs exist as distinct subpopulations and show M2 polarization, scRNA-seq revealed that M1- and M2-associated genes can be highly expressed in the same single cell in early NSCLC, which reflects the plasticity of TAMs, suggesting that M1-like and M2-like functional signatures are not exclusive (98). This study also found no significant difference in the intensity of M2-like signaling in tumors regardless of survival outcome, which further raises doubts about the dogma of an immune suppressive role of M2 macrophages (98). Notably, although M2 did not show a suppressive role, M1 (hot), defined by the top 25th percentile of the M1 marker gene CXCL9’s expression level, was shown to be highly associated with good prognosis, and studies have shown that M1 (hot) TAMs aggregate antitumor T_RM_s *via* CXCL9 expression (98). Overall, scRNA-seq revealed that TAM M2 polarization in the TME appears to be the main pathway for myeloid reprogramming (86), and M1 (hot) is associated with a better prognosis in NSCLC.

TRMs, a subgroup of macrophages that permanently reside in lung tissue, are key cellular components of the immune system in tissues with substantial heterogeneity, having an important role in tissue homeostasis and control of inflammation (99). TRMs were recently shown to provide a protumorigenic niche for early NSCLC cells (34, 89). María et al., using scRNA-seq, found that TRMs drive tissue remodeling programs and tumor cell invasion in early tumor stages. The number of TRMs was significantly reduced compared to that of normal lung tissue with tumor progression in the TME, whereas MDMs predominated in advanced tumors, suggesting that TRMs redistribute in the TME during tumor growth (89). Moreover, the exhaustion of TRMs impaired the ability of early tumor cells to survive and grow, while an increased number of CD4^+^ and CD8^+^ effector T cells, as well as a decrease in PD-1 expression, were found in TRM exhausted lesions (89). These results suggest a protumor role of TRMs in early-stage tumors. In addition, a specific interaction between TRMs and cancer cells in the ovarian cancer microenvironment has been found to promote metastatic spread of ovarian cancer (100). Interacting molecular pathways may represent new therapeutic targets for the treatment of invasive metastatic disease.

##### DCs

DCs excel in antigen presentation so that T and B cells can be activated and play a key role in the induction of anti-tumor T cell immunity. Bone marrow DCs in humans are defined by CD141 and CD1C expression as two “traditional” or “classical” subsets of DCs (101). ScRNA-seq analysis has shown that DCs are phenotypically remodeled and exhibit heterogeneity and a dysfunctional state in the TME, with a possible tendency toward immunosuppression (34, 43). ScRNA-seq analysis revealed that tumor-infiltrating DCs can be divided into multiple heterogeneous DC subgroups (11, 34, 65). One cluster with high expression of CD207, CLEC9A and XCR1 levels may represent CD141^+^ DC, which are associated with the formation of TLS (102) and were significantly lower in LUAD tissue than in normal lung (34, 65). This may be related to its immune tolerance. Another cluster expressed higher levels of CD1c, CX3CR1, and IRF4, possibly representing CD1C^+^ DC. Compared to CD141^+^ DC, CD1C^+^ DC expressed higher levels of CCL22 and CCL17 (34), which is reported to be the chemokine that recruits Tregs. Therefore, it is speculated that CD1C^+^ DCs may create an immunosuppressive TME by recruiting Tregs. Notably, although DCs have antitumor effects, a type of plasmacytoid DC (pDC), which exhibit an immunosuppressive phenotype, was identified in the TME (11, 34, 65, 103). They are enriched in tumor tissue and metastatic lymph nodes (43). These cells can create an immunosuppressive microenvironment, which may lead to a decrease in the efficiency of tumor antigen presentation. Multiregion scRNA-seq analysis revealed that spatial field patterns were evident in DC subsets. In the pDC subpopulation, the spatial enrichment of FOS, FOSB and JUN was increasingly distant from the tumor (41), indicating its pro-tumor effect.

##### Neutrophils

Neutrophils are the first line of defense for our immune system and circulate in the peripheral blood. Neutrophils have chemotactic, phagocytic and bactericidal properties. Studies have shown that neutrophils play an important role by interacting with tumors and immune cells in tumor progression (104). Although there are few scRNA-seq studies on neutrophils, the available findings show that neutrophils are significantly heterogeneous and that the number of cells decreases with tumor progression in NSCLC. ScRNA-seq clustered neutrophils into five subpopulations in NSCLC, forming a continuous state of differentiation with different subpopulations between patients, and neutrophils derived from blood and tumors differ significantly in gene expression (11). In early LUAD, neutrophils are equally abundant in tumor lesions (34). Neutrophil depletion in advanced LUAD may be associated with the slow growth of advanced tumors (70), supporting a protumor role at the late stage (104). However, while most studies have demonstrated the protumor effects of neutrophil, some studies have shown that the direct cytotoxicity of neutrophil can inhibit tumor progression and metastasis (105, 106). For example, circulating neutrophils inhibit T cell proliferation by releasing different molecules. Reactive oxygen species (ROS) and arginase 1 are two of the most widespread inhibitory factors of neutrophil origin (107, 108). To explain this, it has been proposed that the pro- and antitumor effects of neutrophils may be closely related to tumor stage, with antitumor effects in early stages and pro-tumor effects in late stages (109). Combined with scRNA-seq studies of the microenvironment, we hypothesize that neutrophils have an anti-tumor effect in the early stages and may develop into a heterogeneous subpopulation of cells with pro-tumor effects in the late stages due to phenotypic remodeling influenced by the immune microenvironment. However, whether this is the case remains to be further investigated.

#### Phenotypic Shaping of Natural Killer (NK) Cells in the TME of Lung Cancer

NK cells are innate lymphocytes. Its activation is driven by a balance between activating and inhibiting signals and can perform cytotoxic functions in the absence of the major histocompatibility complex (MHC). Therefore, NK cells are a promising tool in cancer immunotherapy. NK cells in the TME of lung cancer are heterogeneous, and their functions are impaired. The proportion of NK cells was found to be reduced through scRNA-seq (41, 43, 86), and with increasing tumor proximity, it was shown a gradual decrease in NK cells, of which were depleted in the LUADs (41, 65). It was the least abundant immune cell lineage in early LUAD lesions. The subset of NK cells expressing CD16 in tumors was significantly reduced (34), indicating that NK cell cytotoxicity is reduced. Researches have shown that the immunosuppressive effects of TME contribute directly to the decrease in NK cell viability. multiple factors in TME converge to regulate NK cell metabolism, such as IL-6, IL-10, transforming growth factor beta (TGF-β), and they alter the balance between NK cell activation and inhibitory signaling, which is a decisive step in NK cell activation, leading to a decrease in their viability (110). Therefore, remodeling the TME to reinvigorate NK cells may be one of the strategies for the treatment of NSCLC. It was found that HIF-1α (hypoxia inducible factor 1 subunit alpha) deficient tumor-infiltrating NK cells have significant antitumor activity (111). Analysis of HIF-1α deficient tumor-infiltrating NK cells in NSCLC through scRNA-seq revealed the presence of an activated NK subpopulation characterized by IκBζ (inhibitor of nuclear factor kappa B zeta) expression, upregulation of NF-κB (nuclear factor kappa B) activity and high functional capacity (112). And the potent antitumor activity and control of tumor progression by HIF-1α deficient NK cells depends on IL-18 from bone marrow cells in the TME (112). These results suggest that a combined approach of IL-18 supply with NK-specific HIF-1α targeting is expected to improve the therapeutic strategy for NK cell-sensitive solid tumors. There was No PD-1 signaling detected in NK cells, but the TIM-3 level is higher *via* scRNA-seq (113). In advanced NSCLC, NK cells are divided into two subgroups. Among them, CD16^+^ subgroups show upregulated expression of transcription factors involved in the cytotoxic function of lymphocytes, while CD16^-^ subgroups highly express tissue resident markers (70), which indicates that NK cells may promote antitumor functions in advanced NSCLC.

### CAFs

CAFs are common stromal cell types that can secrete a variety of provascular factors in the TME. Fibroblasts are heterogeneous in NSCLC (**Figure 5A**). The cluster of fibroblasts was found to be divided into seven known cell types by scRNA-seq in NSCLC (43, 54), with COL13A1^+^ and COL14A1^+^ fibroblasts being the major fibroblast types in early NSCLC tissues (43). Moreover, myofibroblasts were found to replace fibroblasts in the TME (43, 95), which may promote extensive tissue reconstruction (114), angiogenesis (115), and tumor progression (116). Myofibroblasts were characterized by expression of both fibroblastic marker genes, such as PDGFRA and LUM, and smooth muscle marker genes, such as MYLK and ACTA2 (95). And high expression of genes related to myogenesis (MEF2C, MYH11 or ITGA7), the Notch pathway and angiogenesis were found in the highest ACTA2 expression clusters, indicating that these cells were activated (54).

Studies have shown that fibroblasts in the microenvironment can regulate extracellular matrix synthesis and remodeling and angiogenesis, which can promote tumor progression or immunosuppression. Each of the fibroblast types expresses unique collagen and other extracellular matrix molecules (54). Fibroblasts in normal tissues express high elastin and low levels of certain collagens (collagen types I, III, V and VIII) but not others (e.g., collagen type VI) (54), while subpopulations of myofibroblasts exclusively existing in tumor tissues exhibit high expression of collagens such as COL3A1, COL5A1, COL5A2 and COL6A3, other matrix proteins and matrix degrading enzymes (95), suggesting roles in extracellular matrix remodeling. *Via* scRNA-seq, Myofibroblasts in tumor tissue also exhibit high activity of transforming growth factor beta (TGF-β) and JAK/STAT signaling as well as hypoxia-induced pathways (54, 95). PDGF signaling was found to be activated between tumors and CAFs in advanced LUAD (70). Though scRNA-seq, a CAF subpopulation characterized by high expression of genes encoding extracellular matrix proteins is revealed to drive immunotherapy resistance through cellular crosstalk increasing protein levels of PD-L1 and CTLA-4 in Treg cells (117). These expressions are associated with angiogenesis and extracellular matrix biosynthesis and remodeling. The results of studies focusing on CAFs suggest that CAFs may influence tumor development by regulating angiogenesis and extracellular matrix synthesis and remodeling, suggesting that CAFs are an emerging target of antitumor.

### ECs

ECs, also known as angiogenic cells, are involved in forming the lining of blood vessels and constitute a selective barrier between blood and tissue. ECs play an important role in tumor growth, infiltration, and metastasis (118). Therefore, high infiltration of ECs in tumors is often associated with poor prognosis.

ECs show significant heterogeneity in TME (**Figure 5B**). Through scRNA-seq, ECs clustered into multiple heterogeneous subgroups in NSCLC (43, 54), and tumor-specific subpopulations were also identified in addition to the presence of normal tissue subpopulations. For example, a type I (H3) and type II (H4) alveolar capillary EC phenotype was detected, as well as two new capillary phenotypes possibly induced by tumor cytokines in NSCLC (119). Interestingly, in contrast to the extensive phenotypic heterogeneity of vascular ECs, the gene expression profiles of tumor-infiltrating lymphatic vessel ECs and peritumor lymphatic vessel ECs were highly similar, and no subpopulation of lymphatic vessel ECs was detected (119). Capillary ECs in tumors express higher levels of genes involved in MHC II-mediated antigen presentation than other phenotypes, but the costimulatory molecules CD80 and CD86 were not detected (120), suggesting a function as semiprofessional antigen-presenting cells.

Through scRNA-seq, strong activation of angiogenic signals was detected in ECs. Wu found activation of the CXCL12-CXCR4 pathway between tumors and sprouting ECs and strong VEGF signaling interactions between tumors and various types of ECs (70), Kim identified a tumor-derived EC group by scRNA-seq, present in lung and brain metastasis samples, and it displayed strong activation of VEGF and Notch signaling (43), which regulates the development and cell fate determination of ECs (121). In LUAD, angiogenic markers, such as VWA1, HSPG2 and INSR, are highly expressed in tumor endothelial cells consisting of two plasmacytic clusters (95). These findings suggest upregulation of gene expression associated with EC generation in the TME, supporting therapeutic strategies targeting the antiangiogenic pathway in lung cancer and brain metastases. Myc targets were the most enriched signals in tumor-associated ECs, and total read counts in tumor EC clusters were two- to fourfold higher than those in normal EC clusters, indicating that the tumor EC transcription rate is increased (54, 65). This result may be related to Myc enrichment in tumors, as Myc can upregulate transcription (122), identifying a potential vulnerability of tumor ECs to Myc inhibition. However, angiogenic signaling in ECs is decreased in ground glass nodular LUAD (123), which may be related to their inactive state.

Echoing the strong activation of angiogenic signaling, scRNA-seq showed that ECs may suppress the immune response in NSCLC. For example, through scRNA-seq, the expression of genes involved in immune activation and immune cell homing was found to be downregulated in NSCLC, and the expression of genes involved in antigen presentation and chemotaxis was decreased, suggesting that tumor ECs are remodeled to decrease their antigen presentation and immune cell homing activities, thus contributing to tumor immune tolerance (43, 54). Another study identified MLX and MAF as candidate transcription factors for differential gene expression in lymphatic ECs in NSCLC by applying SIENIC, while the expression of Fos/Jun and ELF3 was downregulated, which seemed to be associated with tumor-specific EC phenotype, indicating that Fos/Jun loss underlies the reduced immune stimulatory phenotype of tumor ECs (54). These suggest that promotion of angiogenesis and suppression of antitumor immunity are two mechanisms by which ECs promote lung cancer cell growth.

## Discussion and Conclusions

Over the past period of time, there has been a tremendous shift in the understanding of tumors from being viewed as homogeneous entities to understanding the TME as a heterogeneous ecosystem with single-cell resolution. The use of scRNA-seq which provides high-resolution insights into the intra-tumor heterogeneity and transcriptional activity of cells in the TME reveals that the TME can be regarded as a harbor for tumor promotion and immune suppression. In this review, we depict the microenvironmental profile of lung cancer obtained from scRNA-seq technology, focusing on the phenotypic shaping of stromal cells in the TME. In general, compared to normal lung tissue, tumor lesion sites are enriched with Tregs, depleted CD8+ T cells, B cells especially plasma cells, M2 macrophages, pDCs and depleted of NK cells, and blast B cells. We also discuss the characteristics of various types of stromal cells in the TME based on scRNA-seq data, the heterogeneous expression profile and the mechanisms involved in immunosuppression. These differences may act synergistically to promote an immunosuppressive TME, thereby promoting tumor progression and tolerance to antitumor immunity.

How to reverse immunosuppression is an urgent problem facing lung cancer treatment. The current study concluded that immunosuppression may be the result of a dual effect of extrinsic factors - an increase in the number of immunosuppressive cells such as Tregs and MDSCs and intrinsic factors, including the selection of antigen-deficient tumor variants that limit the antitumor immune response (124). Thus, suppression of immunosuppressive cells and establishment of antitumor immune surveillance may show better results. For example, one study reversed the immunosuppressive TME by promoting the polarization of macrophages from the M2 to the M1 through nanoparticles, showing better therapeutic results (125, 126). Although some progress has been made in reversing immunosuppression, the TME is far more complex than we realized, and targeting multiple rather than just one target in the TME may be the future trend in reversing immunosuppression. Microenvironmental heterogeneity promotes tumor evolution and is a key determinant of tumor biology, therapeutic response, and patient survival (127). Therefore, it is critical to comprehensively characterize the phenotypes and interactions of the various cell types present in tumors. Analyzing the TME, specifically sequencing spatially or temporally distinct tumor regions has begun to reveal a dazzling degree of diversity within tumors. Thus, quantitative measurements of heterogeneity are important for our understanding of microenvironmental heterogeneity to guide the treatment of lung cancer. Recently, techniques have been developed to quantitatively measure the heterogeneity of the solid TME along three spatial dimensions, called the tumor ecosystem diversity index (EDI) and the Shannon diversity index was used to quantify the number of cell types in the microenvironment, and unsupervised Gaussian mixture clustering was used to examine global differences in cell diversity between regions to calculate the EDI score (128). It has been initiated in grade 3 breast cancer and high microenvironmental diversity was found to be associated with poor prognosis, independent of tumor size or genomic characteristics (128).

Distant metastasis is a major cause of death from lung cancer, but specific aspects of metastatic lung cancer and its associated microenvironments remain poorly understood. Analysis of early and metastatic lung cancer by scRNA-seq, Treg cells and exhausted CD8^+^ T cells persisted in both early and metastatic samples, providing a suppressive mechanism of anti-tumor immunity during tumor progression and metastasis (43). B cells were increased in both samples, and lymph node metastases were enriched in germinal center B cells, indicating activation of humoral immune responses (43). Anti-inflammatory macrophages were identified in metastatic lymph nodes, with a subpopulation expressing macrophage inflammatory factors (MIF, CXCL3, and CCL20. and MIF-expressing macrophages also expressed IL1B and TNF at levels comparable to those of pro-inflammatory monocytes, suggesting a unique macrophage profile in metastatic lymph nodes (43). These results suggest that metastatic stage cells are present in varying degrees in almost all primary tumors. In another scRNA-seq of brain metastases and cerebrospinal fluid (CSF), a cluster of cells with elevated cell cycle characteristics in T cells and TAMs was identified, indicating their active proliferation (129). The rest of the assays yielded similar results, suggesting that analysis of CSF can provide critical information about brain TME in a relatively non-invasive manner, avoiding intracranial surgery. Notably, the ratio of CD8/CD4^+^ T cells was significantly higher in the tumors compared to the cerebrospinal fluid (129).

As a newly developed potential technology, the application of scRNA-seq will help us to develop more meaningful new strategies in the diagnosis and treatment of lung cancer. In studying cancer cells, in addition to revealing their heterogeneity, the application of scRNA-seq can help to identify molecular drivers of tumor development. For example, in a study of 28 cells from a mouse model *via* scRNA-seq, Xiong et al. found that these cells all contained the same IGFBP7^R45P^ mutation, suggesting that these cells may have come from a common ancestor, which also suggests that the IGFBP7^R45P^ mutation may be an important driver mutation in lung cancer progression (130). In addition, scRNA-seq also can use to find more meaningful targets for treatment. Park et al. analyzed scRNA-seq data from *in vitro* and *in vivo* and found that TERT and MET were strongly expressed in lung cancer cells (131), suggesting that these two genes could serve as promising cancer biomarkers. Moreover, scRNA-seq also provide new insights into drug resistance of tumor therapy. Kim et al. performed scRNA-seq studies on cells isolated from xenograft tumors from patients with LUAD and found that subpopulations of tumor cells containing KRAS^G12D^ mutations may play an important role in antitumor therapy resistance (132). Similarly, scRNA-seq can be used for analysis in tumor metastasis. In a scRNA-seq study focusing on circulating tumor cells isolated from the CSF of lung cancer patients, high expression of two markers, CEACAM6 and SCGB3A2, was shown (133), suggesting that the expression of these two proteins may be associated with the metastasis of lung cancer.

Several recent studies have shown that scRNA-seq can also be used for prognostic prediction of lung cancer. Multiple ligand-receptor pairs were identified and analyzed based on differential expression of scRNA-seq database in LUAD, and ITGB4, CXCR5 and MET were found to significantly affect the accuracy of prognostic judgments in the prediction model (134). In addition, the construction of a risk score model using the gene co-expression network detected by scRNA-seq can also be used as a tool to predict patient prognosis, and activation of the T cell suppressor p53 signaling pathway and regulatory T cells was found in the high-risk Score subtype, which may contribute to the poorer prognosis of this subtype (135). Some studies explored the relationship between tumor cell stemness index and stemness-related genes and patient prognosis through scRNA-seq and found that mRNA stemness index was higher compared with normal lung tissue in LUAD, and advanced patients exhibited higher mRNAsi and poorer overall survival (136, 137). Therefore, scRNA-seq can be used to predict the prognosis of lung cancer patients by further analysis and study of differentially expressed proteins and genes, etc. Of course, the progress is not limited to these. For example, scRNA-seq revealed that LUSC has higher inter- and intra-tumoral heterogeneity than LUAD (70), while solid LUAD has greater heterogeneity than ground glass nodular LUAD (123).

These findings confirm that scRNA-seq is a powerful tool to resolve the complexity of lung cancer and its microenvironment. However, scRNA-seq still has some limitations. First, the cost of scRNA-seq is expensive. Although the cost has decreased substantially with the development of emerging technologies, its expense still hinders its large-scale application. Moreover, there are still some problems in linking transcript data differences to cell type and function. It is unclear to what extent the transcript differences obtained by scRNA-seq reflect actual cell types and functions. In addition, most scRNA-seq methods are restricted to detecting mRNAs with poly(A) tails [poly(A) + RNAs] only, while mRNAs without poly(A) tails, such as circRNAs and microRNAs, also have an important role, but scRNA-seq technology for these molecules is still insufficient. Although it has limitations, scRNA-seq will hopefully escape these limitations and be increasingly used in the future with time and technological advances.

## Author Contributions

ZJH, SCY and YXY wrote the manuscript. ZJH and SCY wrote the first draft of the manuscript. YXY revised the manuscript and edited. TY edited the manuscript. All authors contributed to the article and approved the submitted version.

## Funding

This work was supported by grants from the academic leader projects of economic and social development in Northwest Liaoning province (No. 10159-1002-2).

## Conflict of Interest

The authors declare that the research was conducted in the absence of any commercial or financial relationships that could be construed as a potential conflict of interest.

## Publisher’s Note

All claims expressed in this article are solely those of the authors and do not necessarily represent those of their affiliated organizations, or those of the publisher, the editors and the reviewers. Any product that may be evaluated in this article, or claim that may be made by its manufacturer, is not guaranteed or endorsed by the publisher.
